# Improved Waste Heat Management and Energy Integration in an Aluminum Annealing Continuous Furnace Using a Machine Learning Approach

**DOI:** 10.3390/e25111486

**Published:** 2023-10-26

**Authors:** Mohammad Andayesh, Daniel Alexander Flórez-Orrego, Reginald Germanier, Manuele Gatti, François Maréchal

**Affiliations:** 1 Industrial Process and Energy Systems Engineering, École Polytechnique Fédérale de Lausanne EPFL, 1950 Sion, Switzerland; francois.marechal@epfl.ch; 2 Department of Energy, Politecnico di Milano, 20156 Milan, Italy; manuele.gatti@polimi.it; 3 Novelis Switzerland SA, 3960 Sierre, Switzerland; reginald.germanier@novelis.adityabirla.com

**Keywords:** annealing continuous furnace, decarbonization, computational fluid dynamics, machine learning, exergy analysis, energy integration

## Abstract

Annealing furnaces are critical for achieving the desired material properties in the production of high-quality aluminum products. In addition, energy efficiency has become more and more important in industrial processes due to increasing decarbonization regulations and the price of natural gas. Thus, the current study aims to determine the opportunities to reduce energy consumption in an annealing continuous furnace and the associated emissions. To this end, the heat transfer phenomenon is modeled and solutions for the decreasing fuel consumption are evaluated so that the overall performance of the process is enhanced. A heat transfer model is developed using the finite difference method, and the heat transfer coefficient is calculated using machine learning regression models. The heat transfer model is able to predict the heat transfer coefficient and calculate the aluminum temperature profile along the furnace and the fuel consumption for any given operating condition. Two solutions for boosting the furnace exergy efficiency are evaluated, including the modulation of the furnace temperature profiles and the energy integration by the recycling of exhaust flue gases. The results show that the advanced energy integration approach significantly reduces fuel consumption by up to 20.7%. Sensitivity analysis demonstrates that the proposed strategy can effectively reduce fuel consumption compared with the business-as-usual scenario for a range of sheet thicknesses and sheet velocities.

## 1. Introduction

The global aluminum market is expected to grow annually by 5.8%, stimulated by an increasing demand for aluminum products, such as sheets and coils, in the automotive industry. Aluminum alloys have low density, good corrosion resistance, a high strength-to-weight ratio and good ductility [1,2]. For these reasons, aluminum is the second most used metal in the modern economy, finding applications not only in transportation sector but also in packaging and buildings [3]. Aluminum alloys are also widely used in aircraft components and structures [4]. Another advantage of the aluminum is its high recyclability, which makes it a sustainable choice for many applications. In fact, the increase in aluminum recycling rates has gained renewed interest, considering that primary (pure) aluminum production has a CO_2_ emission intensity of around 17.1 t_CO2_/t_Al_ [5].

Annealing is a critical process in the manufacture of aluminum coils, as it relieves concentrated stresses that have been introduced during the rolling process and modifies the microstructure of aluminum to improve the material strength, toughness, and corrosion resistance. In this way, it also increases its ductility, which allows the material to be formed and shaped more easily. It is achieved by heating the aluminum coil to a given temperature below the melting point, holding the temperature for a pre-defined time, and finally cooling it down either with water or air. The quality of the finished product is improved and the risk of defects is reduced. Complex microstructure evolutions including static re-crystallization, phase transformation, and a change in crystal orientation, grain morphology and size happen during the heat treatment of the aluminum coils [6]. In this regard, the precise heat transfer and band transportation processes in the annealing continuous line (ACL) ensures the efficient and reliable heating rates that comply with the expected production quality and throughput.

The carbon footprint and the production cost of the aluminum coils strongly depend on the energy efficiency of the ACL furnace. Therefore, it is necessary to develop an accurate operational model for predicting and improving the furnace performance and energy consumption. Computational fluid dynamics (CFD) has been widely applied as a powerful tool for analyzing the heat transfer and fluid flow in heat treatment furnaces [7,8,9,10,11,12]. A three-dimensional CFD model simulates the heat and mass transfer through the specified domain by numerically solving the governing equations in discrete zones called finite volumes. However, one disadvantage of the CFD simulations is the high computational time. Hajaliakbari and Hassanpour [13] applied a numerical approach based on the finite volume method to calculate the energy efficiency of an annealing continuous furnace in the steel industry. According to the authors, both of strip velocity and heating power should be carefully adjusted in each heating schedule. Strommer et al. [14] developed a first-principle model that relies on mass and energy balances to describe the dynamic behavior of the furnace. Although the model differs from measurements, it is suitable for real-time applications of control due to the moderate computational effort. Cho et al. [15] proposed a data-driven neural network MPC (model predictive controller) as a fast predictive model for the real-time control of an ACL furnace. He et al. [16] developed a first-principle model to determine the strip temperature using the heat balance method. The model inputs are the strip dimensions and zone temperature, and it provides the strip temperature distribution in the furnace. Differently from the configuration of the ACL furnace studied in the present work, the annealing furnaces of previous studies rely on temperature-resistant rolls for transporting the band and mostly use a radiant tube to supply the heat. In this work, both the forced convection and radiation heating processes and the levitation force for lifting and transporting the aluminum band are driven by the hot flue gases injected through the furnace nozzles. This contactless transportation system leads to a higher quality for the final product.

The application of machine learning methods to model and predict heat transfer phenomena in thermofluid systems has drawn the attention of researchers in order to reduce the computational time related to CFD simulations. Supervised machine learning can be used to improve the understanding of the heat transfer processes by developing accurate models for predicting heat transfer coefficients. Kwon et al. [17] the applied random forest algorithm to predict the heat transfer coefficient for convection in a cooling channel integrated with variable rib roughness. Accordingly, compared with simple analytical correlations, machine learning regressors can be much more accurate, especially for unsteady, nonlinear systems. Mehralizadeh et al. [18] developed several machine learning models to predict the boiling heat transfer coefficient of different refrigerants in finned-tube applications and compared them with existing empirical correlations. The models predict the heat transfer coefficient for the test data with good agreement. Yoo et al. [19] used machine learning to predict the heat transfer coefficient for condensation in the presence of non-condensable gas, in terms of the total pressure, mass fraction of the non-condensable gas, and wall subcooling. According to the authors, outside of the application range, the existing correlations do not accurately predict the heat transfer coefficient. Thus, a machine learning technique was applied to better predict the heat transfer coefficient for other operating conditions based on new experimental results. In the present work, four supervised learning algorithms are used to predict the heat transfer coefficient of the aluminum furnace using relevant operating conditions as the model inputs. Thus, the application of machine learning models may help in improving the operational efficiency and reliability of ACL furnaces.

In view of this, major cost savings could be achieved by implementing enhanced waste heat recovery approaches, thanks to reduced fuel consumption, lower risk perception, and mitigation of the environmental impact. Some authors have studied ways of recovering waste heat energy in the aluminum industry. Senanu et al. [20] studied the effect of flue gas recycling from aluminum electrolysis cells with a CO-to-CO_2_ converter to chemically recover waste heat. Jouhara et al. [21] designed a heat-pipe heat exchanger for recovering waste heat from a thermal treatment furnace. Brough and Jouhara [22] highlighted the relevant potential for waste heat recovery in the aluminum production processes and reviewed different sources of waste heat and applicable technologies. Flórez-Orrego et al. [23] conducted a systemic study on decarbonization processes in the aluminum remelting industry to elucidate opportunities for enhanced waste heat recovery and renewable energy integration.

In contrast to previous studies, in which waste heat recovery was performed by recuperation, in the present work, two solutions are proposed and analyzed for improving the waste heat recovery and furnace efficiency. The first solution deals with the adoption of optimal temperature profiles that guarantee the lowest exergy loss for each one of the furnace zones. The adjustment of the temperature of each zone to a suitable level that still ensured the heat transfer rate proved to be a thermodynamically efficient way to distribute the energy requirement among different zones while reducing the stack loss. The second approach consists of thermally integrating the different zones of the ACL furnace, as in certain zones the roof gases may still have enough energy to preheat the aluminum band in the colder zones. Currently, each zone temperature is controlled by a number of fired heaters and the waste heat available in the stack gases is used for preheating hot water distribution networks at low temperature. In this regard, the first solution could be highly compatible with the decarbonization strategy via electrification of the heat supply, which may halve the emissions of the aluminum industry provided that electricity from renewable resources is available [24]. The second solution can be adopted in the case of biomass integrated gasification approaches, as the amount of waste heat released in the gasifier and other reactors could be harvested to preheat the combustion air of syngas-fired ACL furnaces.

In this study, computational fluid dynamics (CFD) tools are used for modeling and simulating different operating conditions of the ACL furnace and retrieve data that characterizes the thermodynamic and fluid dynamic performance. After the pre-processing of the experimental and CFD data, four machine learning models are trained and their accuracies for predicting the overall heat transfer coefficients are evaluated. Moreover, the operating model quantifies the natural gas consumption and exergy losses. Next, strategies for fuel reduction, e.g., energy integration, are proposed and analyzed to improve the energy efficiency of the ACL furnace. Sensitivity analysis is also conducted to verify the effect of aluminum sheet thickness and transport velocity on the energy integration results.

The novelty of this work relies on the development of a fast and accurate machine-learning-based tool able to predict the operation of an actual ACL (annealing continuous line) furnace. To the best authors’ knowledge, previous studies deal with the application of computational fluid dynamic models for specific case studies and, thus, they cannot be applied to predict the heat transfer parameters for the other operating conditions of the furnace. This work represents a novel solution that can be applied in real plants using a model predictive control for adjusting the heat treatment process according to the real-time performance. Secondly, some strategies for fuel reduction targeting are proposed and analyzed using the developed model considering the quality of the energy flows (namely, exergy), which has not been analyzed in previous studies. In this regard, the analysis of different potential heating profiles allows us to determine the best temperature profile in the furnace that guarantees the lowest irreversibility of the whole energy system.

## 2. Description of the ACL Furnace Operating Principle

**Figure 1:**
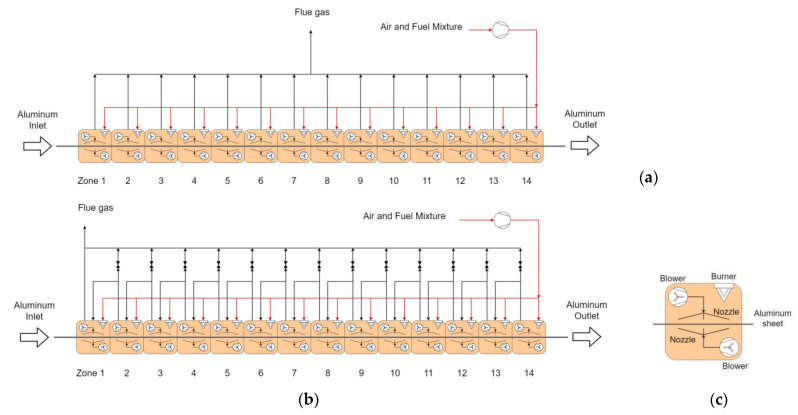
Process flow diagrams of ACL furnaces considering (**a**) separate exhausts and direct discharge to environment and (**b**) integrated configuration for recycling exhaust gas. (**c**) Schematic view of each zone.

The unrolled aluminum coil enters the furnace at 20 °C and moves through the fourteen furnace zones (see Figure 1) at constant speed in order to achieve an annealing temperature of around 500–600 °C. The aluminum must remain at high temperature for a specific duration, which varies from recipe to recipe and is typically confidential. Each zone contains three nozzles at the top and three nozzles at the bottom that are fed by two recirculation fans on each side. Natural-gas-fired burners provide the heat duty. The aim of the nozzles is twofold, namely, to redirect the hot gases towards the heating strip of the aluminum and support its mass, so that the material never touches the furnace internals and the integrity of the treated surface is maintained. The furnace is 42 m long, and the typical values for aluminum speed range from 30 to 40 m/min. The nominal thickness can vary from 1 to 2.5 mm. For higher production throughputs, sheet velocity can be increased up to 45 m/min.

Differently from the layout shown in Figure 1a, wherein the flue gases from the stack are discharged directly into the environment, in the proposed integrated configuration (see Figure 1b), the exhaust gas of a hotter zone can be sent to a colder zone in order to capitalize on the waste heat still available in the flue gases. The heat integration approach using recycling is further analyzed to quantify the reduction in fuel consumption. In order to operate within practical conditions, a limitation is considered on the volumetric flowrate of the recycled flue gas. In other words, to maintain the space velocity of the hot gases inside each zone, the maximum volumetric flowrate of each zone in the integrated configuration should be no higher than in the configuration of Figure 1a, i.e., without recycled exhaust gases. In this way, the overall effect of the energy integration will be the maximization of the waste heat recovery from hot exhaust gases to preheat the colder zones, whereas the balance of the energy requirement is achieved using conventional fired heaters.

## 3. Methodology

A combination of computational fluid dynamics simulations and machine learning models is used to predict the heat transfer coefficient for different operating conditions of the ACL furnace. The input parameters are selected as the velocity and the thickness of the aluminum sheet, the gas temperature, and the fan recirculation percentage. This approach allows determining important energy transport parameters with a low computational time, in comparison to the execution of a complete CFD simulation. By applying the energy balance, the temperature profile of the aluminum sheet along the whole furnace can be outlined, as well as the waste heat available in the ACL stack.

**Figure 2:**
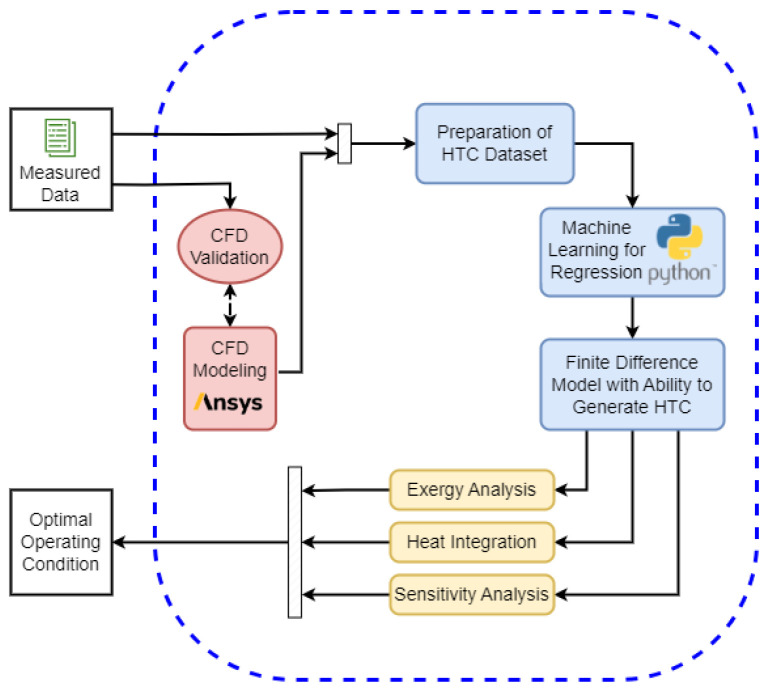
Schematic of the methodology used to validate the CFD calculation using experimental data, prepare the simulated dataset, perform the regression on a simplified heat transfer model, and calculate the energy integration performance in the ACL furnace.

Figure 2 summarizes the procedure used to develop and apply the proposed energy integration approach and hierarchize the different temperature profiles that ensure the minimum exergy destruction rate. Measured data, whenever available, are used to validate the computational fluid dynamic simulations performed in ANSYS Fluent^®^ 2022 R2 software. Further details on the mathematical models and solvers used in the CFD simulation are discussed in the next section. After the computational results are validated, the data is extracted and prepared to be fitted to the regression model. The regression model consists of a simplified finite different representation of the heating process of the aluminum sheet. The derivation of this simplified model is described in the subsequent sections. It is worth noting that, differently from an oversimplified lumped model, which does not consider the lag between the internal and external temperature profiles of the aluminum sheet, the proposed simplified model relying on finite difference discretization can capture the inertia of the aluminum heating and the delay in the heat diffusion from the surface to the center of the heated material. After the data is regressed on the simplified model using polynomial regression machine learning method in Tensorflow/Keras libraries of Python programming language, the model is able to predict the heat transfer coefficient and it can be used to calculate both energy and exergy balances, and perform the recycling and sensitivity analyses, as shown in Figure 2.

### 3.1. Configuration of the Computational Fluid Dynamics Model

**Figure 3:**
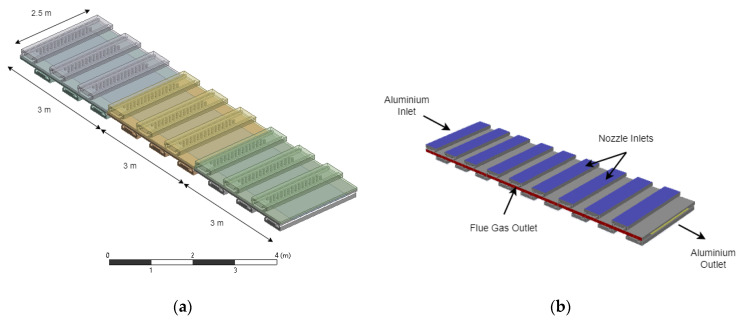
CFD simulation in ANSYS Fluent^®^. (**a**) Control volume, which includes three zones. (**b**) Boundaries conditions of the domain.

The control volume adopted for the CFD simulation is based on three zones of the furnace, as shown in Figure 3. Since the setup parameters used in the simulation can be adjusted to analyze either the frontend or backend zones of the furnace, a sample volume allows reducing the computational time while keeping the accuracy of the solution. A Cartesian meshing is applied with an inflation mesh near the most critical heat transfer and flow surfaces, namely, the nozzle and aluminum faces. The simulation setup considers the activation of the energy equation for coupled heat transfer between the aluminum sheet and the hot gases. A k-ω SST turbulence model is selected to represent the perturbation and eddies present in the highly non-laminar flow. Additionally, the P1 radiation model is considered. Since the aluminum coil is continuously unrolled and passed at a constant speed through the different zones in the ACL furnace, the CFD simulation considers a constant motion for the aluminum sheet. The governing equations of the simulations performed in ANSYS Fluent^®^ [25,26] can be summarized in Equations (1)–(5):

- Mass conservation equation:

(1) $$\nabla .\left(\rho \vec{v}\right)=0$$

- where $\vec{v}\;\left(m/s\right)and\;\rho \;(\frac{kg}{m^{3}})$ are gas velocity vector and density, respectively.

- Momentum conservation equation:

(2) $$\nabla .\left(\rho \vec{v}\vec{v}\right)=-\nabla p+\nabla .\left(\overset{̿}{\tau }\right)+\rho \vec{g}$$

where *p* (N/m^2^), $\overset{̿}{\tau }$ (N/m^2^), and $\rho \vec{g\;}$ (N/m^3^) are static pressure, the stress tensor, and the gravitational body force, respectively.

- Transport equations for the SST *k*-ω model:

The turbulence kinetic energy (*k*) and the specific dissipation rate (ω) are obtained from the following transport equations:

(3) $$\frac{\partial }{\partial t}\left(\rho k\right)+\frac{\partial }{\partial x_{i}}\left(\rho ku_{i}\right)=\frac{\partial }{\partial x_{i}}\left(\Gamma_{k}\frac{\partial k}{\partial x_{i}}\right)+{\tilde{G}}_{k}-Y_{k}\;\frac{\partial }{\partial t}\left(\rho \omega \right)+\frac{\partial }{\partial x_{i}}\left(\rho \omega u_{i}\right)=\frac{\partial }{\partial x_{i}}\left(\Gamma_{\omega }\frac{\partial \omega }{\partial x_{i}}\right)+G_{\omega }-Y_{\omega }+D_{\omega }$$

In these equations, ${\tilde{G}}_{k}$ represents the generation of turbulent kinetic energy due to mean velocity gradients. $G_{\omega }$ is the generation of ω. $\Gamma_{k}$ and $\Gamma_{\omega }$ represent the effective diffusivity of *k* and ω, respectively. $Y_{k}$ and $Y_{\omega }$ are the dissipation of k and ω due to turbulence.

- Energy equation:

(4) $$\nabla .\left(\vec{v}\left(\rho E+p\right)\right)=\nabla .\left(k_{eff}\nabla T-h\vec{J}+\left({\overset{̿}{\tau }}_{eff}.\vec{v}\right)\right)$$

where *k_eff_* (W/mK) is the conductivity, *T* (K) is the temperature, *h* (J/kg) is sensible enthalpy, and $\vec{J}$ (kg/m^2^s) is the diffusion flux. The three terms on the right-hand side of the equation correspond to energy transfer due to conduction, species diffusion, and viscous dissipation, respectively.

- The P-1 model equations:

(5) $$-\nabla .q_{r}=\alpha G-4\alpha n^{2}\sigma T^{4}$$

where α is the absorption coefficient (-), *G* (W/m^2^) is the incident radiation, and σ is the Stefan–Boltzmann constant (5.67 × 10^−8^ W m^−2^ K^−4^). The expression for $-\nabla .q_{r}$ is directly substituted into the energy equation to account for heat sources due to radiation.

### 3.2. The Simplified Heat Transfer Model Used to Apply Supervised Learning Techniques

A simplified model of the heat transfer process in the ACL furnace was developed in order to apply supervised learning regression techniques to the data obtained from the CFD simulations and validated using experimental runs. In this way, a metamodeling approach allows calculation of the heat transfer coefficient given a number of operating conditions and, consequently, determining the temperature profile of the aluminum along the zones of the ACL furnace. The exergy loss and fuel consumption can be also determined based on the overall energy balance of the system, including stack losses.

**Figure 4:**
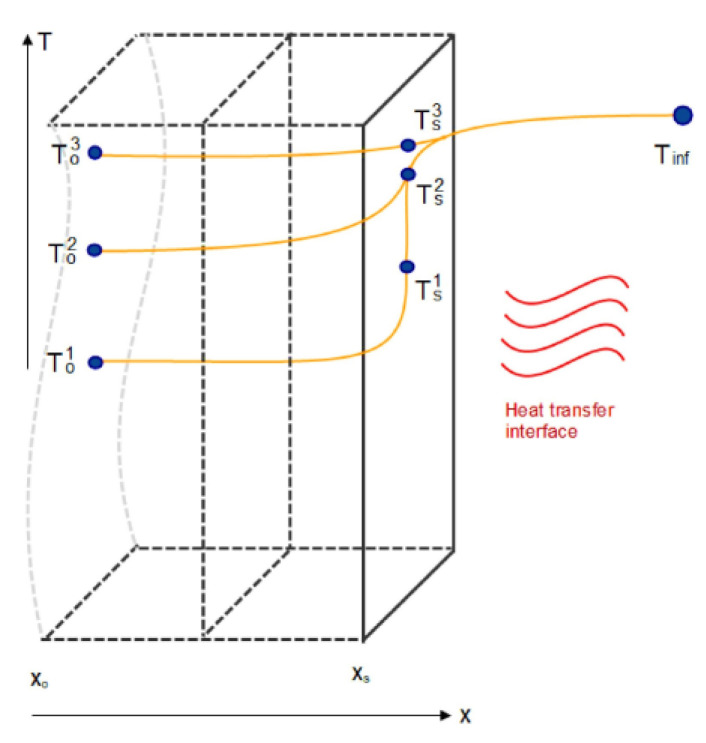
Schema of the derivation of the explicit finite difference-based discretization approach used to apply supervised machine learning to the experimental and simulation data.

Using the finite differences method (FDM), the simplified model concept can be devised in such a way that the gas–solid and the internal solid heat transfer phenomena is represented in one superficial and one inner point of the aluminum material, respectively (Figure 4). In other words, it is assumed that the heat diffusion towards and the energy accumulation inside the aluminum body (_o_) occur within a given time lapse thanks to continuous radiative and convective heat transfer from the hot gas (_inf_) to the aluminum surface (_s_). In this way, the temperature variation of the internal mass can be differentiated from that of the aluminum surface, which is contrary to other approaches that impose lumped models [27] with a given time constant and consider the internal aluminum temperature as equal to the superficial temperature. According to Figure 4, the explicit finite differences-based discretization of the differential energy balances given in Equations (6) and (8) for the aluminum inner body (T_o_) and surface (T_s_), respectively, results in Equations (7) and (9). The aluminum band is discretized along the length of the furnace and Equations (7) and (9) are applied for each cell to determine the aluminum temperature (T_o_) profile along the length of the furnace. Controlling this has a significant impact on the achievement of the heat treatment requirements.

For the aluminum inner body (at *T_O_*):

(6) $$kA\frac{dT}{dx}=\rho AdxC_{p}\frac{\partial T}{\partial t}$$

(7) $$kA\frac{\left(T_{S}\left(t_{1}\right)-T_{O}\left(t_{1}\right)\right)}{\left(x_{S}-x_{O}\right)}=\rho A\frac{{(x}_{S}-x_{O})}{2}C_{p}\frac{\left(T_{O}\left(t_{2}\right)-T_{O}\left(t_{1}\right)\right)}{\left(t_{2}-t_{1}\right)}$$

For the aluminum surface (at *T_s_*):

(8) $$-kA\frac{dT}{dx}+HTC\;A\left(T_{\infty }-T_{S}\right)=\rho AdxC_{p}\frac{\partial T}{\partial t}$$

(9) $$-kA\frac{\left(T_{S}\left(t_{1}\right)-T_{O}\left(t_{1}\right)\right)}{\left(x_{S}-x_{O}\right)}+HTC\;A\left(T_{\infty }\left(t_{1}\right)-T_{S}\left(t_{1}\right)\right)=\rho A\frac{{(x}_{S}-x_{O})}{2}C_{p}\frac{\left(T_{S}\left(t_{2}\right)-T_{S}\left(t_{1}\right)\right)}{\left(t_{2}-t_{1}\right)}$$

**Figure 5:**
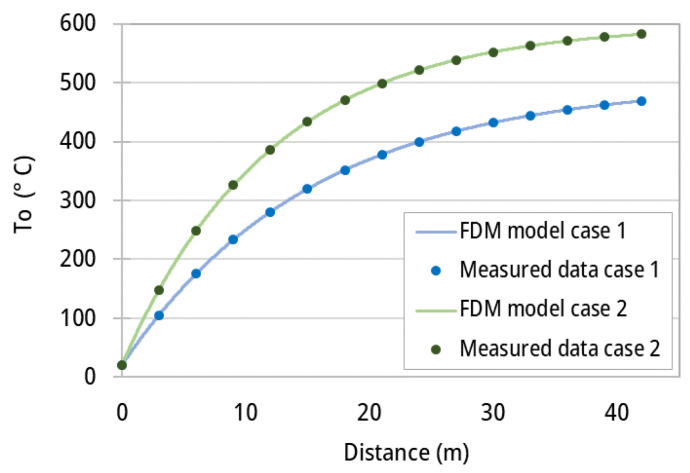
Simplified finite difference-based model fitting on the experimental data points of aluminum temperature (T_o_) along the ACL furnace length.

Since the unknown total heat transfer coefficient is required to determine the temperatures along the ACL furnace in a transient regime and for various operational conditions; supervised learning techniques are used to regress the data gathered from CFD simulations and experimental runs and to predict the heat transfer coefficient [28]. To this end, the explicit finite difference-based discretization model is used to fit the known operating conditions, such as gas temperature, fan power percentage, and aluminum temperature, to the unknown heat transfer coefficient (*HTC*) in Equation (9). Figure 5 depicts two cases of the FDM model fitting on experimental data where the inner aluminum temperature (T_o_) profile along the length of ACL furnace is fitted on the measured temperatures to determine the corresponding *HTC*. This procedure is conducted on all the experimental data to form a dataset of HTCs. Afterwards, four types of supervised learning algorithms, namely linear, polynomial, decision tree, and random forest are trained based on the dataset to predict the *HTC* for arbitrary operating conditions.

### 3.3. Exergy Loss Calculation Based on the Energy Balance on the ACL Furnace

In order to determine the fuel consumption in the whole ACL furnace, a zone-wise energy balance can be calculated, as shown in Equation (10):

(10) $$Q_{Al,z}+Q_{FG,z}+Q_{wall,z}+Q_{leakage,z}=Q_{fuel,z}+Q_{recirculation,z}\;\mathrm{for}z\;=\;1–14$$

where *Q_Al,z_* is the amount of energy that is effectively absorbed by the aluminum sheet (kW); *Q_FG,z_* is the energy leaving the ACL furnace with the flue gas (kW); and *Q_wall,z_* and *Q_leakage,z_* are the heat dissipation through the furnace walls and the leakage losses (e.g., hot gas leakage), respectively. *Q_fuel,z_* and *Q_recirculation,z_* are, respectively, the energy input with the fuel consumed and with the heat recovered from recycled flue gases produced at a downstream (hotter) zone (e.g., for heat integration). All the terms in the energy balance of Equation (10) can be explicitly represented as in Equation (11) if no combustion air preheating is adopted:

(11) $$\begin{array}{c} Q_{Al,z}+\left(\alpha +1\right)\cdot {\dot{m}}_{F,z}\cdot C_{p,FG,z}\cdot \left(T_{FG,z}-T_{ref}\right)+U\cdot A\cdot \left(T_{wall,z}-T_{amb}\right)+\xi_{z}{\dot{\cdot m}}_{F,z}\cdot LHV\;={\dot{m}}_{F,z}\cdot LHV+{\dot{m}}_{RC}\cdot C_{P,FG,z}\cdot \left(T_{FG,z+1}-T_{FG,z}\right) \end{array}\mathrm{for}z\;=\;1–14$$

where α is the mass air-to-fuel ratio (kg_air_/kg_fuel_); *U* is the heat transfer coefficient at the furnace walls (W/m^2^K); ξ is the percentage of energy loss due to hot gas leakage (-); LHV is the lower heating value of the fuel (kJ/kg); *C_p,FG_* is the heat capacity of the flue gases (kJ/kgK); m_F_ and m_RC_ are the mass flows of the fuel and the recycled gases from a hotter zone (kg/s); *T_wall_* and *A* are the temperature (K) and the external surface area (m^2^) of the furnace walls; *T_FG_*, *T_ref_*, and *T_amb_* are the flue gases (K), reference (298 K), and ambient temperatures (298 K); and *T_FG,z_* and *T_FG,z_*_+1_ are the temperatures of the recycled hot gases at the current and a next (hotter) zones. Rearranging Equation (11), the rate of fuel consumed per zone (kg/s) can be calculated using Equation (12):

(12) $${\dot{m}}_{F,z}=\frac{Q_{Al,z}+U\cdot A\cdot \left(T_{wall,z}-T_{amb}\right)-{\dot{m}}_{RC,z}{\cdot C}_{P,FG,z}\cdot (T_{FG,z+1}-T_{FG,z})}{\left(1-\xi_{z}\right)\cdot LHV-\left(\alpha +1\right)\cdot C_{P,FG,z}\cdot \left(T_{FG,z}-T_{ref}\right)}$$

Different mechanisms of exergy destruction occur inside the ACL furnace. Expectedly, combustion is the most irreversible phenomenon; however, its impact can only be mitigated either by reducing the amount of fuel consumption (e.g., better heat recovery and isolation) or avoiding highly irreversible diffusion and heat transfer mechanisms between the combustion gas species. The latter is technically challenging, unless electrical heating powered by an ideal van ’t Hoff fuel cell supersedes combustion technology. Another important source of exergy destruction is the heat transfer rate at a finite temperature difference between the hot gases and the aluminum sheet. This contribution to the exergy destruction can be calculated using Equation (13) for each zone:

(13) $${Ex}_{dest-HT,z}=Q_{Al,z}\cdot \left(1-\frac{T_{amb}}{{\bar{T}}_{FG,z}}\right)-Q_{Al,z}\cdot \left(1-\frac{T_{amb}}{{\bar{T}}_{Al,z}}\right)=Q_{Al,z}\cdot \left(\frac{T_{amb}}{{\bar{T}}_{Al,z}}-\frac{T_{amb}}{{\bar{T}}_{FG}}\right)$$

where *T_amb_* is the dead state temperature (298 K); *T_Al_* and *T_FG_* are aluminum and hot gases temperatures (K), respectively; and *Q_al_* is the heat transferred from the hot gas to the aluminum sheet (kW). The other irreversibility mechanisms are the losses associated with the hot gas leakage, the flue gases leaving the stack at hot temperatures (e.g., if heat integration is not or only partially implemented), and the exergy destruction via wall heat losses. These exergy destruction rates can be calculated based on Equations (14)–(16), respectively:

(14) $${Ex}_{dest,Leakage,\;\;z}=\xi_{z}{\dot{\cdot m}}_{F,z}\cdot \varphi \cdot LHV$$

(15) $${Ex}_{dest,StackGas,z}= [\left(\alpha +1\right)\cdot {\dot{m}}_{F,z}\cdot C_{p,FG,z}\cdot \left(T_{FG,z}-T_{ref}\right)-{\dot{m}}_{RC}\cdot C_{P,FG,z}\;\cdot \left(T_{FG,z}-T_{FG,z-1}\right)]\cdot \left(1-\frac{T_{amb}}{{\bar{T}}_{FG,z}}\right)$$

(16) $${Ex}_{dest,Wall,z}=UA\left(T_{wall,z}-T_{amb}\right)\left(1-\frac{T_{amb}}{{\bar{T}}_{FG,z}}\right)$$

where φ is the ratio between the chemical exergy and the LHV of the fuel (b^CH^/LHV ~ 1.02) and ${\bar{T}}_{FG}$ is the logarithmic mean temperature of the flue gases calculated as ${\bar{T}}_{FG}=\left(T_{FG}-T_{amb}\right)/ln\left(T_{FG}/T_{amb}\right)$. Note that in Equation (15), the energy available in the flue gases of the current zone (*z*) can still be recycled and used to preheat the aluminum sheet in a previous zone (*z* − 1), thus reducing the total amount of energy rejected in the flue gases of the current zone. It is worth noticing that although the calculation of the heat flowing through the ACL furnace walls uses the furnace wall temperature, the exergy loss must be calculated using the actual temperature of the hot gases inside the furnace. This approach aims to include the exergy destruction along the isolation layer.

**Figure 6:**
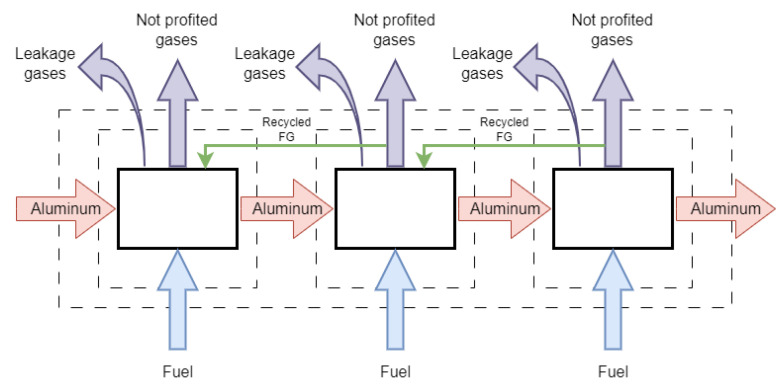
Schema of the calculation of the zone-wise exergy destruction and the total exergy destruction in the ACL. The dash lines depict the zones and total control volumes.

Finally, a zone-wise and total exergy destruction can be calculated in the furnace considering the exergy inflows and outflows (Figure 6):

For the total exergy destruction ${Ex}_{dest,total}$, Equation (17):

(17) $${Ex}_{Al,\;in}-{Ex}_{Al,\;out}+\sum_{1..14}{Ex}_{fuel,\;z}={Ex}_{dest,\;total}$$

For the zone-wise exergy destruction ${Ex}_{dest,z}$, Equation (18):

(18) $${Ex}_{Al,\;in,z}-{Ex}_{Al,\;out,z}+{Ex}_{fuel,\;z}-{\dot{m}}_{RC}\cdot C_{P,FG,z}\cdot \left(T_{FG,z}-T_{FG,z-1}\right)\left(1-\frac{T_{amb}}{{\bar{T}}_{FG,z}}\right)={Ex}_{dest,z}$$

where the exergy supplied by the fuel and the exergy recovered by the aluminum are calculated by using Equations (19) and (20), respectively:

(19) $${Ex}_{fuel,\;\;z}={\dot{m}}_{F,z}\cdot \varphi \cdot LHV$$

(20) $${Ex}_{Al,\;in,z}-{Ex}_{Al,\;out,z}=Q_{Al,z}\cdot \left(1-\frac{T_{amb}}{{\bar{T}}_{Al,z}}\right)$$

## 4. Results and Discussion

In this section, the CFD model validation is presented. The performance evaluation of the machine learning algorithms is discussed in the light of statistical indicators that measure the goodness of regression. Next, improvements for waste heat management and energy integration are analyzed by energy and exergy analysis in the ACL furnace. Finally, a sensitivity analysis is applied to estimate the variation in the fuel consumption as a function of the aluminum sheet velocity and thickness.

### 4.1. Validation of CFD Model

**Figure 7:**
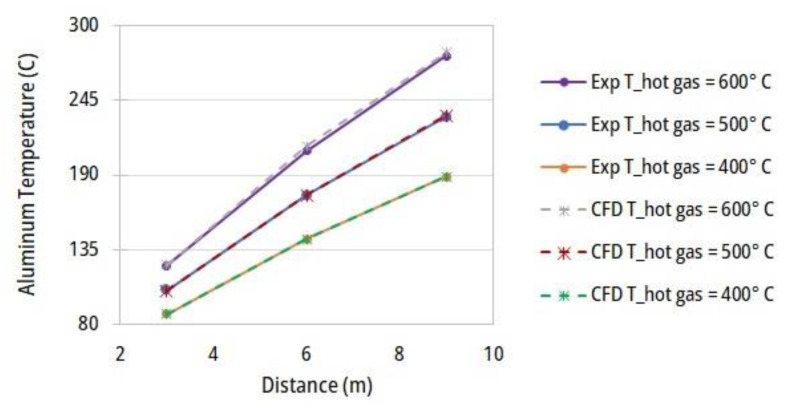
Validation of the simulated (CFD) temperature profiles along the ACL furnace.

A computational fluid dynamic modelling and simulation of the ACL furnace is applied in the current study and this model is calibrated and validated using different experimental data available for certain operating conditions (T_hot gas_ = 500 °C). Then, after the calibration, the error between the CFD and experimental data are calculated for two cases of T_hot gas_ = 400 and 600 °C. The maximum error of the temperature profile is equal to 0.9%. Figure 7 depicts the aluminum temperature at three first zones for three constant profiles of hot gas temperatures. The temperature variations predicted using the CFD model (dashed lines) show good agreement with the measured data (solid lines).

### 4.2. Implementation of Supervised Learning Algorithms to Predict the Heat Transfer Coefficient

**Figure 8:**
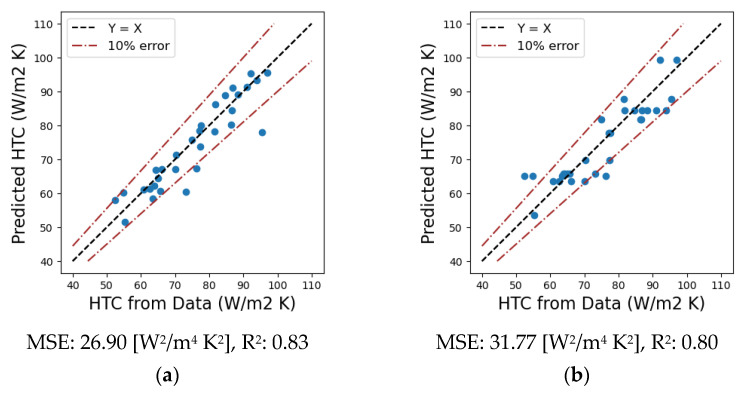

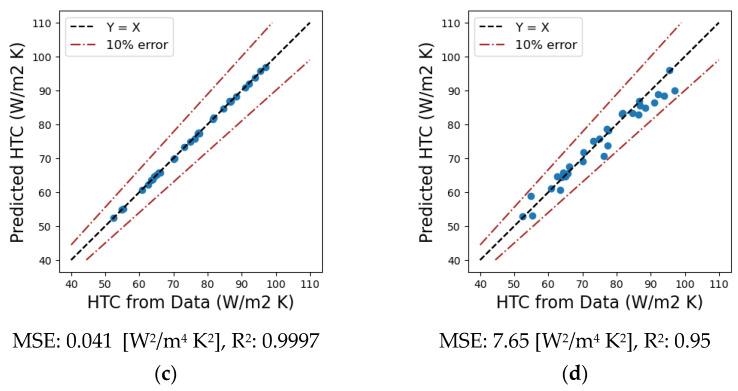
Performance metrics of the test predictions for the heat transfer coefficient using (**a**) linear, (**b**) decision tree, (**c**) polynomial, and (**d**) random forest regression models.

After the CFD and experimental data of the ACL furnace are processed, the simplified model based on the finite differences discretization approach is used to regress the heat transfer coefficient (*h*) as the output variable for different input operating conditions. To this end, four machine learning regression models, namely linear, polynomial, decision tree, and random forest, are applied to the dataset, which is divided into two subsets, namely training and testing data. The performance of the regression algorithms is checked by calculating different statistical metrics, such as the mean squared error (MSE) and coefficient of determination (R^2^). The results of the metrics evaluation are presented in Figure 8. Accordingly, the polynomial regression model shows the best performance when predicting the heat transfer coefficient. Next, the heat transfer coefficient is predicted and used together with the simplified finite difference-based discretized model to calculate the aluminum temperature profiles for any given operating condition. The model inputs arethe aluminum thickness and band speed, percentage of recirculation fan, and furnace gas temperature.

### 4.3. Energy Input and Exergy Destruction as Functions of the Temperature Profile in the ACL Furnace

As expected, the higher the temperature of the hot gases, the higher the energy loss via stack and leakage gases and through the furnace walls. In addition, the higher the zone temperatures, the larger the internal exergy losses due to increased finite temperature differences between the hot gases and the aluminum. Thus, rational selection of the roof temperatures may avoid exergy losses (both internal and to the environment) being exacerbated. The zone temperatures could be controlled by using electrically heated elements, by consuming less fuel, or even by recycling hot gases from hotter zones to achieve the heating rates without incurring excessive avoidable exergy losses. This opportunity of optimizing the temperature profiles inside the ACL furnaces is further analyzed.

**Figure 9:**
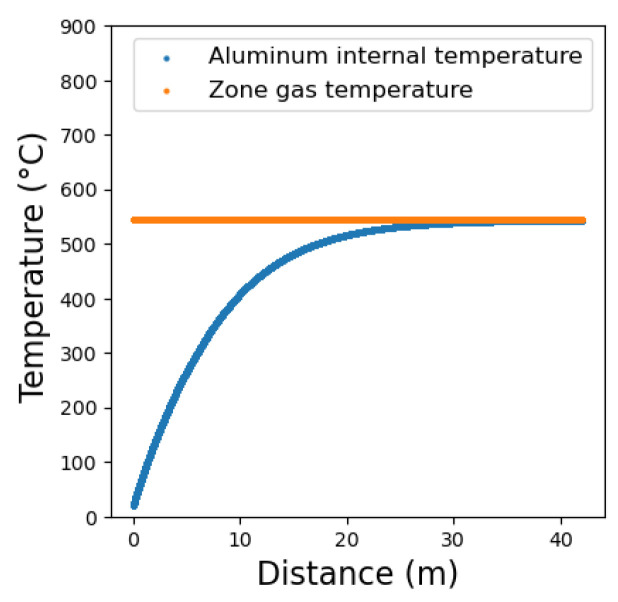
Hot gases and aluminum temperature profiles along the ACL furnace for the business-as-usual scenario (i.e., constant zonal temperatures).

Figure 9 shows the constant hot gas temperature and the increasing aluminum temperature profiles in the business-as-usual (BAU) scenario, in which no temperature variation or waste heat recovery is implemented along the different zones of the ACL furnace. The operational constraints, such as the temperature set point for the continuous annealing process (~500–600° C) and the maintaining time of the treatment process, often defined by planning and material engineers as a recipe, should be observed. The sheet thickness and velocity are set to 1 mm and 30 m/min, respectively. According to Figure 9, there is a large driving force at the initial ACL furnace zones that reduces as the aluminum set point temperature is reached. During the initial zones, the temperatures of the stack and leakage gases are consequently higher, entailing not only larger exergy losses to environment but also avoidable exergy losses due to high finite temperature differences between the hot gases and the aluminum sheet.

**Figure 10:**
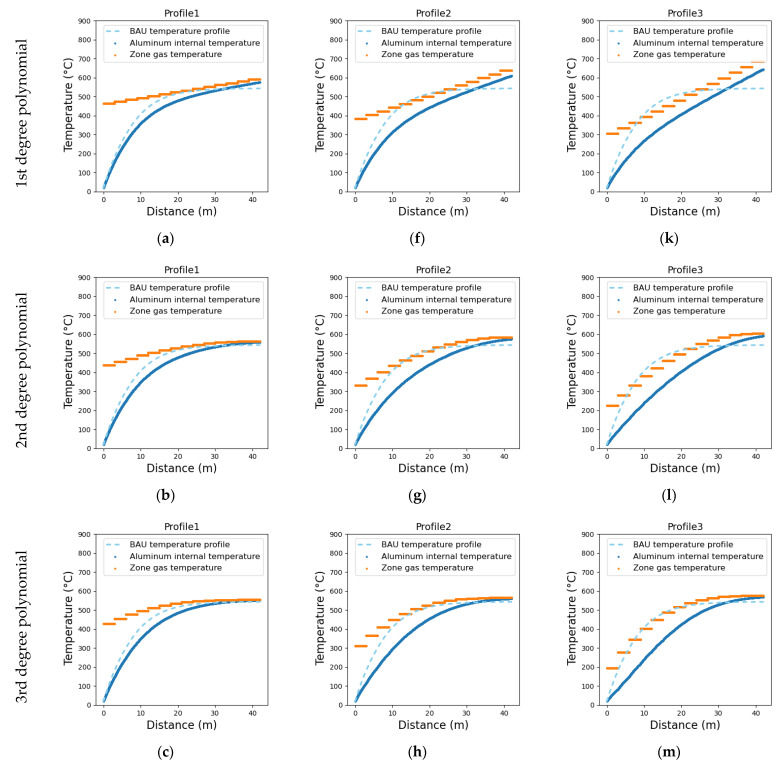

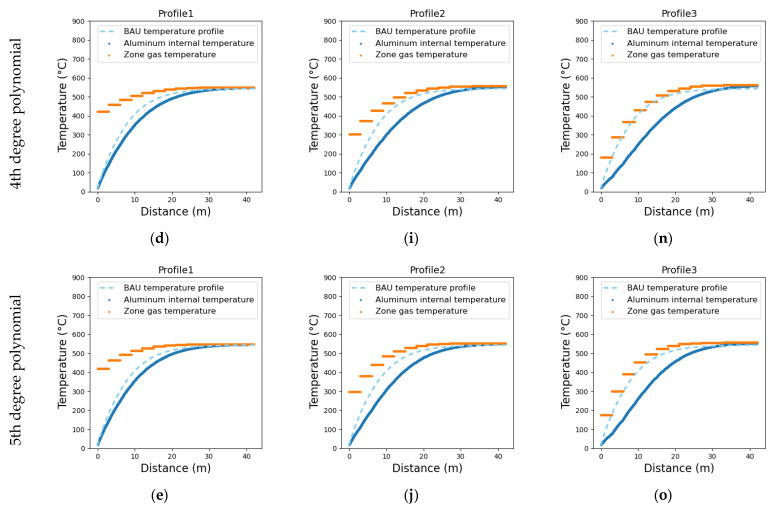
Simulated aluminum (solid blue curves) and roof gas (orange curve) temperature profiles along the ACL furnace zones considering linear and polynomic roof gas temperature profiles in subfigures (**a**–**o**). The aluminum temperature profile for the BAU scenario is shown as a dashed light blue curve. The profiles’ equations (T = a_0_+a_1_ × x+a_2_ × x^2^+a_3_ × x^3^+a_4_ × x^4^+a_5_ × x^5^) are provided in Appendix A.

**Figure 11:**
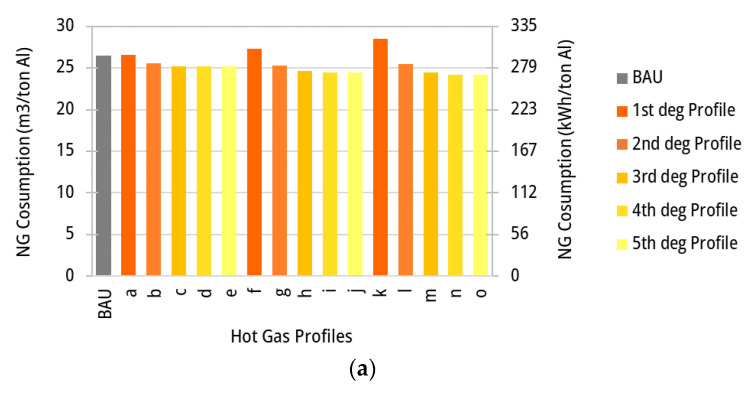

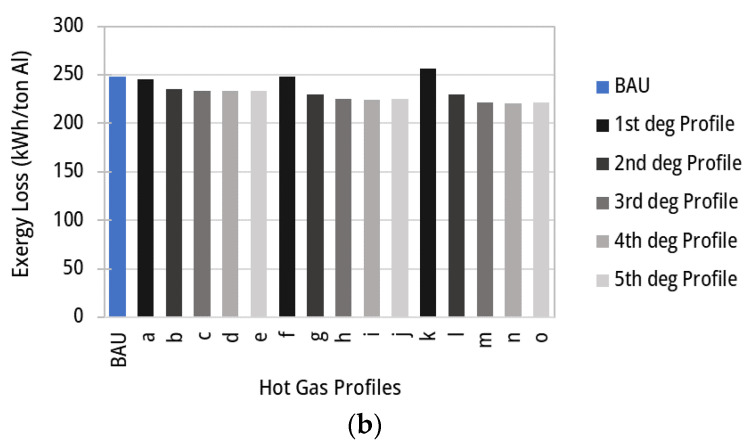
(**a**) Fuel consumption for different temperature profiles of hot gases along the ACL zones and for separate flue gas discharge (no gas recycling). (**b**) Total exergy loss for different temperature profiles of hot gas along the ACL zones and for separate flue gas discharge (no gas recycling).

The fuel consumption and the total exergy losses (internal and to the environment) in the ACL furnace are calculated for the business-as-usual scenario as 26.5 m^3^_NG_/tAl and 248.4 kWh/tAl, respectively. Those values are also determined for other temperature profiles, such as those shown in Figure 10, including different linear and polynomial temperature profiles in the ACL furnace. It is worth noting that energy integration via hot gas recycling is not yet applied, thus the total fuel consumption and process exergy destruction in the ACL furnace (Figure 11a,b, respectively) are initially calculated only as a function of the variation of the temperature profiles over the zones. Accordingly, the profile (o) demonstrates the best outcome, with a decrease in NG consumption of 8.5%, where exergy destruction reduces by 11.0% as the temperature profiles vary along the ACL furnace.

**Figure 12:**
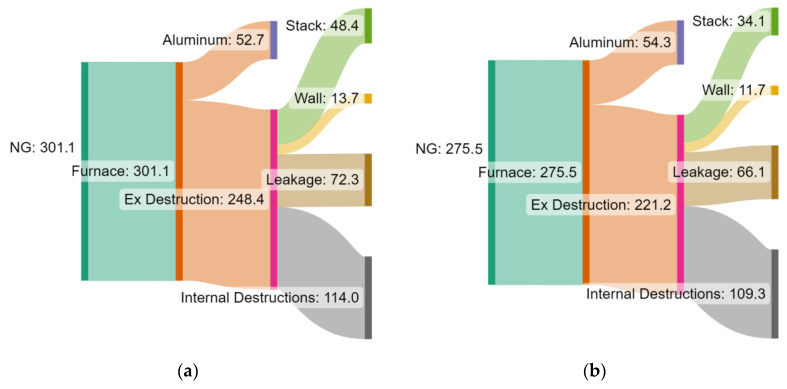
Grassmann diagrams of exergy flows in the ACL furnace (in kWh/tAl) for two representative cases: (**a**) BAU scenario and (**b**) profile (o) (Figure 10), i.e., with separate flue gas discharge (no recycling).

The exergy balance is depicted in Grassmann diagrams in Figure 12 for the two cases of the BAU scenario (Figure 9) and for profile (o) (Figure 10) without gas recycling. The evident variation in the profiles entails a major impact in terms of stack exergy loss reduction (29.5%) since the exhaust gas temperature is much lower in the initial zones of profile (o). For the same reason, considering that the energy and exergy losses are a function of the zone temperatures, the wall exergy destruction decreases by 14.6% when the variation in the hot gas profile is implemented. Moreover, a reduction of 8.6% is observed in exergy loss from leakages. Internal exergy destruction, which includes exergy losses from combustion, heat transfer, and other internal losses, decreases by 4.1% when employing the operational strategy suggested by profile (o) (Figure 10). A lower finite temperature difference between the hot gases and the aluminum reduces the irreversibility associated with the large driving force of the heat transfer phenomena.

**Figure 13:**
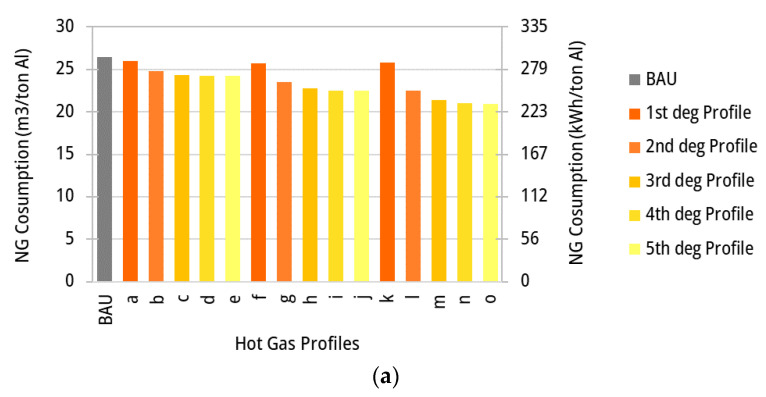

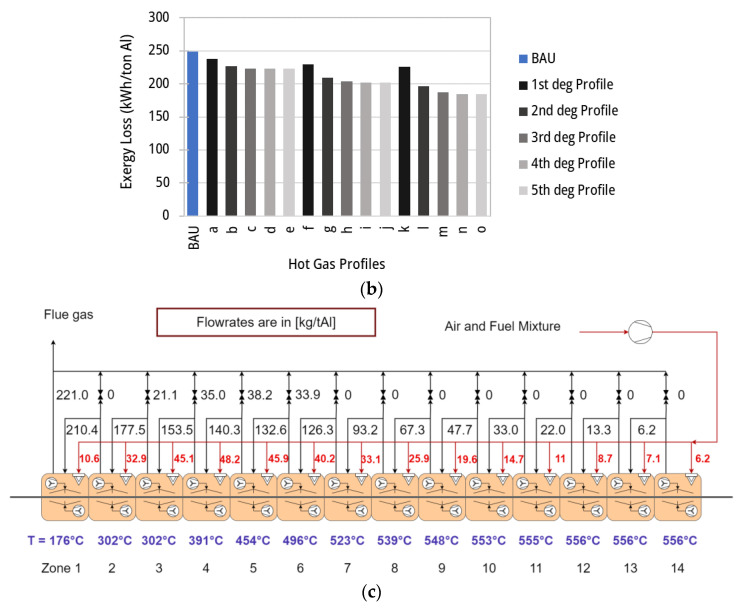
(**a**) Fuel consumption for different temperature profiles of hot gases (Figure 10) along the ACL zones for the energy integrated configuration (recycling enabled). (**b**) Total exergy losses for the different temperature profiles of hot gases (Figure 10) along the ACL zones for the energy integrated configuration (recycling enabled). (**c**) Mass flowrates of the flue gases (kg/tAl) per zone for temperature profile (o) (Figure 10) when the energy integration approach is adopted (recycling enabled).

Clearly, if the temperature of stack hot gases leaving the ACL furnace zones is limited by the maximum temperature attainable by the aluminum sheet, a large share of the hot gases’ exergy may still be available at relatively high temperatures before it is rejected to the environment. Thus, together with the temperature variation over the zones, heat integration via hot gas recirculation can also be a suitable solution for reducing the irreversibility rates by recovering heat from the hot gases leaving hotter, downstream zones to heat the colder zones. This fact, in turn, reduces the fuel consumption. Obviously, waste heat recovery via the recycling of hot stack gases is not as interesting for the BAU scenario as for the variable zone temperature scenario. In the BAU case, energy could still be used to preheat the combustion air, but it will not produce the same effect in terms of decreasing the fuel input, since the exergy input necessary at the highest temperature will always need an additional consumption of fuel to reduce the exergy balance. On the other hand, when the exhaust gas of a zone is sent to a previous zone for heat recovery purposes (see Figure 1b), the potential for waste heat recovery is limited by the maximum temperature of the aluminum sheet; however, the total energy available at a high temperature may still be thermodynamically sufficient to provide the entire heat to the aluminum load. Thus, the amount of energy, but more importantly the quality thereof, plays an important role in the rational energy use and may help issuing recommendations based on the second principle of thermodynamics. The results of the energy consumption (m^3^/tAl) and exergy destruction (kWh/tAl), calculated for the same profiles in Figure 10 but considering the energy integrated approach, are shown in Figure 13a,b, respectively. Those figures could be contrasted with Figure 11a,b to find that the exergy losses decrease when variable temperature profiles along the ACL furnace zones are adopted. Interestingly, it can be argued that the linear temperature profiles of the hot gases may indicate the minimum temperature necessary to achieve the heating process; thus, it could be an ideal candidate for the temperature set points in the zones. However, it can be also observed from Figure 10 that those profiles also impose heating rates that may delay the attainment of the annealing temperatures and thus represent shorter maintaining times at those conditions. However, depending on the recipe adopted by the materials engineers, the profiles will provide the required heating and maintaining rates. In this regard, other temperature profiles are analyzed in the light of the energy integration analysis, so that the effect of those temperature profiles on the reduction in natural gas consumption and exergy losses can be elucidated. For the sake of comparison, heating the aluminum sheet by using a constant temperature profile for hot gases may demand as much as 26.5 m^3^ of natural gas per ton of aluminum, whereas the adoption of a polynomial profile such as that shown in Figure 10o and with heat recovery would only require 21.0 m^3^/tonAl. Thus, the latter profile can save 20.7% of the required fuel and decrease 25.8% of the total exergy losses. Figure 13c shows the mass flowrate of recirculation and exhaust streams for profile (o). A limitation is set on the volumetric flowrate of the recycled flue gas to prevent the stream from flow choking. Due to this limitation, the exhaust gases from zones 3–6 are partially sent to their previous zones and some fractions are discharged through the stack. Lower zonal temperatures (thus, higher gas density) entail higher mass flowrates corresponding to a volumetric limit. As shown in Figure 13c, the recirculation mass flowrate increases from zones 6 to 3.

**Figure 14:**
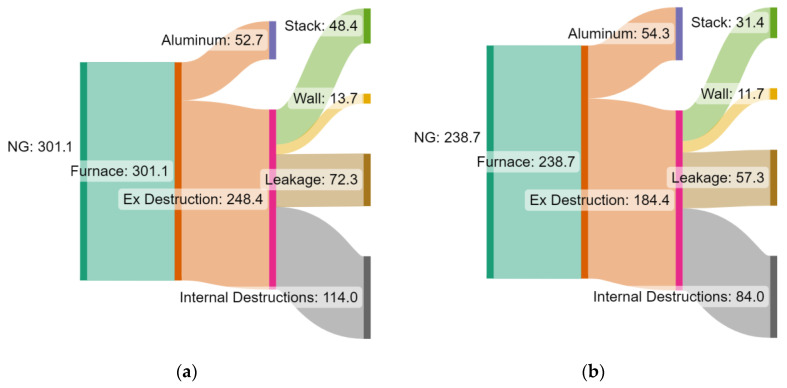
Grassmann diagrams of exergy flows (in kWh/tAl) in the ACL furnace for (**a**) the BAU scenario (same as Figure 12a) and (**b**) profile (o) (Figure 10), i.e., when the energy integration approach is adopted (recycling enabled).

Additionally, the Grassmann diagrams with the exergy flows for both the BAU scenario and the scenario with temperature profile (o) (Figure 10), considering the energy integration approach (i.e., recycling enabled), are depicted in Figure 14a,b, respectively. For the heat integrated configuration, a higher fraction of the total exergy input flows into the aluminum load. Reductions in exergy losses are observed for all the types. The heat recovery from exhaust gases reduces the fuel consumption by 20.7%. As a result, the internal irreversibility due to combustion, leakages, and stack loss is sharply decreased.

### 4.4. Sensitivity Analysis to the Aluminum Sheet Thickness and Velocity

**Figure 15:**
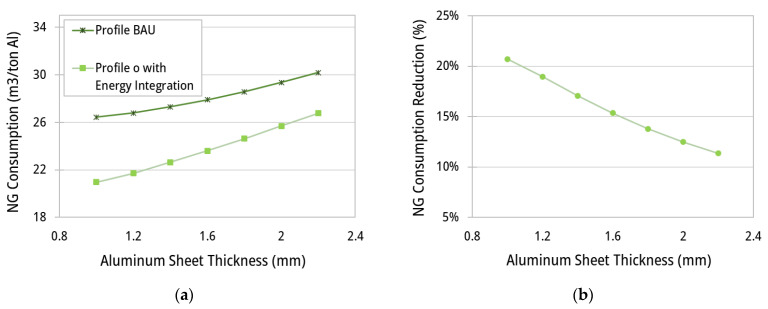
Plots of (**a**) specific fuel consumption (m^3^/tAl) and (**b**) reduction percentages (%) of the scenario with temperature profile (o) (Figure 10), with heat integration (recycling enabled), in comparison with the BAU scenario as a function of the aluminum sheet thickness.

In the previous section, the energy consumption and the exergy destruction in the ACL furnace are calculated to show the effect of the variable profiles of the set-point temperatures for the hot gases and the advantages of the waste heat recovery from the zone stacks. The results showed a significant reduction in fuel consumption for specified values of aluminum sheet thickness (1 mm) and velocity (30 m/min) throughout the ACL furnace. However, the ACL operating conditions may vary depending on customers’ requests and production throughput. Thus, the performance of the proposed energy-saving solutions should be also discussed for a range of aluminum sheet velocities and thicknesses. The specific fuel consumption (m^3^/tAl) and the reduction percentages (%) with respect to the BAU scenario are recalculated for aluminum thickness ranging from 1 to 2.2 mm (Figure 15).

According to Figure 15a, fuel consumption expectedly increases by increasing the sheet thickness. For lower thicknesses, a higher surface-to-volume ratio allows the aluminum to more easily and quickly achieve the treatment temperature and the maintaining time along the ACL furnace. By adopting the constant hot gas profile (BAU scenario), the fuel consumption ranges from 26.5 to 30.2 m^3^/tAl for sheet thicknesses between 1 and 2.2 mm, respectively. On the other hand, the application of a non-constant profile (i.e., profile (o) (Figure 10)) together with enhanced energy integration decreases the fuel consumption by up to 21.0 and 26.8 m^3^/tAl for the same range of thicknesses. Figure 15b depicts the reduction percentages of NG consumption due to the implementation of profile (o) (Figure 10) with heat integration in comparison with the BAU profile. As can be seen, fuel consumption reduces by 20.7 to 11.3% for sheet thicknesses of 1 to 2.2 mm, respectively.

**Figure 16:**
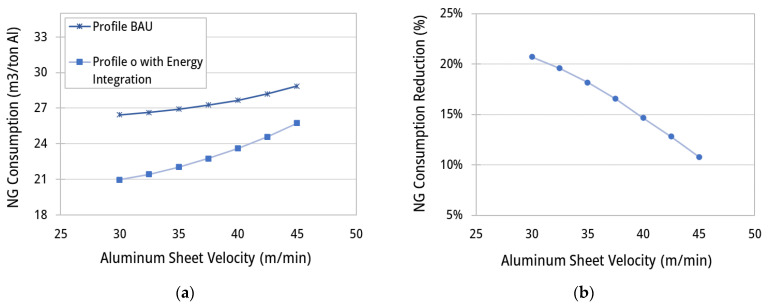
Plots of (**a**) specific fuel consumption (m^3^/tAl) and (**b**) reduction percentages (%) for the scenario with temperature profile (o) (Figure 10), with heat integration (recycling enabled), in comparison with the BAU scenario as a function of the aluminum sheet velocity.

Figure 16 shows the effect of the aluminum sheet velocity on the energy consumption in the ACL furnace, considering the BAU temperature profile and profile (o) (Figure 10). Naturally, fuel consumption increases by increasing the band velocity, as a larger amount of mass of aluminum needs to be processed per unit of time. In cases of higher velocity, it is required that the furnace operates at higher temperatures to provide a higher heat flux for reaching the heat treatment temperatures on time and providing the maintaining times. For temperature profile (o) (Figure 10), the natural gas (NG) consumption varies between 21.0 to 25.7 m^3^/tAl for aluminum velocities of 30 to 45 m/min, respectively. Implementation of improved temperature profiles and energy integration approaches (hot gas recycling) may lead to a 20.7 to 10.8% reduction in fuel consumption for band velocities from 30 to 45 m/min.

## 5. Conclusions

ACL furnaces are utilized for the heat treatment of aluminum sheets in the rolling industry. In the current study, an energy model of this furnace is developed using the finite difference method (FDM) and machine learning (ML) approaches trained on the basis of the experimental data available and the computational fluid dynamics (CFD) simulations in order to characterize the thermal performance of the system and propose solutions for waste heat management. Four ML models are evaluated for regression of the heat transfer coefficient (HTC). The polynomial model shows the best performance, with an MSE of 0.06 (W^2^/m^4^ K^2^) and a coefficient of determination (R^2^) equal to 0.9997. An average error of 0.43% is observed, which is more precise than the models in the open literature (around 5% reported by [13]). This operational model calculates the aluminum temperature profile along the ACL furnace, as well as the fuel consumption, and allows achieving an exergy analysis based on arbitrary operating conditions. A low computational time makes the model suitable for real-time controlling and optimization applications.

Solutions for improvement of the energy performance are also assessed, including the variation in the furnace temperature profiles and the energy integration via the partial recycling of the hot flue gases. The results demonstrate that the heat integration significantly increases the efficiency of the operating conditions for non-constant temperature profiles and also decreases the fuel consumption by up to 20.7% compared with the business-as-usual (BAU) scenario. The sensitivity analysis on ACL fuel consumption for aluminum sheet thickness variation from 1 to 2.2 mm shows an increase in natural gas consumption of 27.6%. Additionally, an increase in the band speed from 30 to 45 m/min leads to a 22.4% increase in fuel consumption. By increasing both parameters, more fuel is required, but the proposed solutions with non-constant temperature profiles in the ACL furnace with energy integration (hot gas recycling) still effectively reduce the fuel consumption and therefore the associated environmental impact.

## Data Availability

Not applicable.

## Nomenclature

**Symbols**	
ρ	Density (kg/m^3^)
$\vec{v}$	Gas velocity vector (m/s)
p	Static pressure (Pa)
$\overset{̿}{\tau }$	Stress tensor (N/m^2^)
$\vec{g\;}$	Gravitational acceleration (m/s^2^)
k	Turbulence kinetic energy (J/kg)
ω	Specific dissipation rate (m^2^/s^3^)
${\tilde{G}}_{k}$	Generation of turbulent kinetic energy (J/kg)
$G_{\omega }$	Generation of specific dissipation rate (m^2^/s^3^)
$\Gamma_{k}$	Effective diffusivity of turbulent kinetic energy (m^2^/s)
$\Gamma_{\omega }$	Effective diffusivity of specific dissipation rate (m^2^/s)
$Y_{k}$	Dissipation of turbulent kinetic energy (J/kg)
$Y_{\omega }$	Dissipation of specific dissipation rate (m^2^/s^3^)
*k*_eff_	Conductivity (W/m K)
T	Temperature (°C)
h	Sensible enthalpy (J/kg)
$\vec{J}$	Diffusion flux (kg/m^2^ s)
α	Absorption coefficient (-)
G	Incident radiation (W/m^2^)
σ	Stefan–Boltzmann constant, 5.67 × 10^−8^ (W/m^2^ K^4^)
$C_{p}$	Aluminum specific heat capacity (J/kg K)
A	Cross-sectional area of aluminum (m^2^)
α	Mass air-to-fuel ratio (kg_air_/kg_fuel_)
U	Heat transfer coefficient of furnace walls (W/m^2^ K)
ξ	Percentage of energy loss due to hot gas leakage (-)
Ex	Exergy (J)
φ	Ratio of the chemical exergy to LHV of the fuel (-)
MSE	Mean squared error
R^2^	Coefficient of determination (-)
**Subscripts**	
_o_	Aluminum inner body
_s_	Aluminum surface
∞	Hot gas
_FG_	Flue gases
_F_	Fuel
_Al_	Aluminum
_RC_	Recycled gases from a hotter zone
_wall_	Furnace walls
_ref_	Reference
_amb_	Ambient
_z_	Current zone
_z+1_	Next (hotter) zone
_dest_	Destruction
_HT_	Heat transfer
**Abbreviations**	
FDM	Finite difference method
ML	Machine learning
HTC	Heat transfer coefficient
NG	Natural gas
LHV	Lower heating value
BAU	Business-as-usual

## Appendix A

The equations of zone temperature profiles shown in Figure 10 are summarized in Table A1.

**Table A1:** The equations of the profiles in Figure 10 (T = a_0_+a_1_ × x+a_2_ × x^2^+a_3_ × x^3^ + a_4_ × x^4^+a_5_ × x^5^). T in °C, x in m.

Profile	Curve Equation
BAU	T = 544.3
a	T = 3.25x + 459.18
b	T = −0.0833x^2^ + 6.75x + 427.81
c	T = 0.0021x^3^ − 0.2596x^2^ + 10.514x + 412.56
d	T = −5.0E−5x^4^ + 0.0089x^3^ − 0.5392x^2^ + 14.558x + 402.6
e	T = 1.0E−06x^5^ − 0.0003x^4^ + 0.023x^3^ − 0.9332x^2^ + 18.898x + 394.73
f	T = 6.5x + 375.25
g	T = −0.1667x^2^ + 13.5x + 311.63
h	T = 0.0043x^3^ − 0.5192x^2^ + 21.029x + 280.41
i	T = −0.0001x^4^ + 0.0178x^3^ − 1.0784x^2^ + 29.117x + 261.59
j	T = 3.0E−6x^5^ − 0.0006x^4^ + 0.0461x^3^ − 1.8665x^2^ + 37.796x + 246.05
k	T = 9.75x + 290.43
l	T = −0.25x^2^ + 20.25x + 195.44
m	T = 0.0064x^3^ − 0.7788x^2^ + 31.543x + 149.67
n	T = −0.0002x^4^ + 0.0266x^3^ − 1.6176x^2^ + 43.675x + 120.09
o	T = 4.0E−6x^5^ − 0.0009x^4^ + 0.0691x^3^ − 2.7997x^2^ + 56.694x + 97.179

## References

[1] Dong H.-R., Li X.-Q., Li Y., Wang Y.-H., Wang H.-B., Peng X.-Y., Li D.-S. (2022). A review of electrically assisted heat treatment and forming of aluminum alloy sheet. Int. J. Adv. Manuf. Technol..

[2] Lee J., Bong H.J., Kim D., Lee Y.-S., Choi Y., Lee M.-G. (2020). Mechanical properties and formability of heat-treated 7000-series high-strength aluminum alloy: Experiments and finite element modeling. Met. Mater. Int..

[3] Majeau-Bettez G., Krey V., Margni M. (2021). What future for primary aluminium production in a decarbonizing economy?. Glob. Environ. Change.

[4] Zhou B., Liu B., Zhang S. (2021). The advancement of 7xxx series aluminum alloys for aircraft structures: A review. Metals.

[5] Deng L., Johnson S., Gencer E. (2022). Environmental-Techno-Economic analysis of decarbonization strategies for the Indian aluminum industry. Energy Convers. Manag..

[6] Gao P., Ren Z., Zhan M., Xing L. (2022). Tailoring of the microstructure and mechanical properties of the flow formed aluminum alloy sheet. J. Alloys Compd..

[7] Mayrhofer M., Koller M., Seemann P., Prieler R., Hochenauer C. (2022). CFD investigation of a vertical annealing furnace for stainless steel and non-ferrous alloys strips—A comparative study on air-staged & MILD combustion. Therm. Sci. Eng. Prog..

[8] Arkhazloo N.B., Bazdidi-Tehrani F., Jadidi M., Morin J.-B., Jahazi M. (2022). Determination of temperature distribution during heat treatment of forgings: Simulation and experiment. Heat Transf. Eng..

[9] Arkhazloo N.B., Bazdidi-Tehrani F., Morin J.-B., Jahazi M. (2021). Optimization of furnace residence time and loading pattern during heat treatment of large size forgings. Int. J. Adv. Manuf. Technol..

[10] Jóźwiak P., Hercog J., Kiedrzyńska A., Badyda K., Olevano D. (2020). Thermal Effects of Natural Gas and Syngas Co-Firing System on Heat Treatment Process in the Preheating Furnace. Energies.

[11] Nave O. (2020). Modification of semi-analytical method applied system of ODE. Mod. Appl. Sci..

[12] Dou R., Zhao H., Zhao P., Wen Z., Li X., Zhou L., Zhang R. (2020). Numerical model and optimization strategy for the annealing process of 3D coil cores. Appl. Therm. Eng..

[13] Hajaliakbari N., Hassanpour S. (2017). Analysis of thermal energy performance in continuous annealing furnace. Appl. Energy.

[14] Strommer S., Niederer M., Steinboeck A., Kugi A. (2014). A mathematical model of a direct-fired continuous strip annealing furnace. Int. J. Heat Mass Transf..

[15] Cho M., Ban J., Seo M., Kim S.W. (2023). Neural network MPC for heating section of annealing furnace. Expert Syst. Appl..

[16] He F., Wang Z.-X., Liu G., Wu X.-L. (2022). Calculation Model, Influencing Factors, and Dynamic Characteristics of Strip Temperature in a Radiant Tube Furnace during Continuous Annealing Process. Metals.

[17] Kwon B., Ejaz F., Hwang L.K. (2020). Machine learning for heat transfer correlations. Int. Commun. Heat Mass Transf..

[18] Mehralizadeh A., Shabanian S.R., Bakeri G. (2020). Investigation of boiling heat transfer coefficients of different refrigerants for low fin, Turbo-B and Thermoexcel-E enhanced tubes using computational smart schemes. J. Therm. Anal. Calorim..

[19] Yoo J.M., Lee D.H., Hong D.J., Jeong J.J. Application of machine learning technique in predicting condensation heat transfer coefficient and droplet entrainment rate. Proceedings of the Transactions of the Korean Nuclear Society Virtual Spring Meeting.

[20] Senanu S., Solheim A., Lødeng R. (2022). Gas Recycling and Energy Recovery. Future Handling of Flue Gas from Aluminium Electrolysis Cells. Light Metals.

[21] Jouhara H., Nieto N., Egilegor B., Zuazua J., González E., Yebra I., Igesias A., Delpech B., Almahmoud S., Brough D. (2023). Waste heat recovery solution based on a heat pipe heat exchanger for the aluminium die casting industry. Energy.

[22] Brough D., Jouhara H. (2020). The aluminium industry: A review on state-of-the-art technologies, environmental impacts and possibilities for waste heat recovery. Int. J. Thermofluids.

[23] Orrego F., Alexander D., Dareen D., Reginald G., François M. A Systemic Study for Enhanced Waste Heat Recovery and Renewable Energy Integration towards Decarbonizing the Aluminium Industry. Proceedings of the 36th International Conference on Efficiency, Cost, Optimisation, Simulation and Environmental Impact of Energy Systems—ECOS 2023.

[24] Teske S., Niklas S., Talwar S. (2022). Decarbonisation pathways for industries. Achieving the Paris Climate Agreement Goals: Part 2: Science-Based Target Setting for the Finance Industry—Net-Zero Sectoral 1.5 °C Pathways for Real Economy Sectors.

[25] Fluent A. (2011). Ansys Fluent Theory Guide.

[26] Zhao J., Ma L., Zayed M.E., Elsheikh A.H., Li W., Yan Q., Wang J. (2021). Industrial reheating furnaces: A review of energy efficiency assessments, waste heat recovery potentials, heating process characteristics and perspectives for steel industry. Process Saf. Environ. Prot..

[27] Kumbhar S.V., Sonage B.K. (2019). Unsteady-state lumped heat capacity system design for tube furnace for continuous inline wire annealing process. Heat Transf..

[28] Hughes M.T., Kini G., Garimella S. (2021). Status, challenges, and potential for machine learning in understanding and applying heat transfer phenomena. J. Heat Transf..

